# Nepali oral microbiomes reflect a gradient of lifestyles from traditional to industrialized

**DOI:** 10.1186/s40168-024-01941-7

**Published:** 2024-11-04

**Authors:** Erica P. Ryu, Yoshina Gautam, Diana M. Proctor, Dinesh Bhandari, Sarmila Tandukar, Meera Gupta, Guru Prasad Gautam, David A. Relman, Ahmed A. Shibl, Jeevan Bahadur Sherchand, Aashish R. Jha, Emily R. Davenport

**Affiliations:** 1 Department of Biology, Pennsylvania State University, University Park, PA USA; 2 Genetic Heritage Group, Program in Biology, New York University Abu Dhabi, Abu Dhabi, UAE; 3 Department of Microbiology and Molecular Genetics, McGovern Medical School, The University of Texas Health Science Center at Houston, Houston, TX USA; 4 Public Health Research Laboratory, Institute of Medicine, Maharajgunj, Kathmandu, Nepal; 5 School of Public Health, University of Adelaide, Adelaide, SA Australia; 6 Organization for Public Health and Environment Management, Lalitpur, Bagmati, Nepal; 7 Sidney Kimmel Medical College, Philadelphia, PA USA; 8 Department of Geography, Tribhuvan University, Nepalgunj, Nepal; 9 Department of Medicine, Stanford University, Stanford, CA USA; 10 Department of Microbiology and Immunology, Stanford University, Stanford, CA USA; 11 Section of Infectious Diseases, Veterans Affairs Palo Alto Health Care System, Palo Alto, CA USA; 12 Center for Genomics and Systems Biology, and Public Health Research Center, New York University Abu Dhabi, Abu Dhabi, UAE; 13 Huck Institutes of the Life Sciences, Pennsylvania State University, University Park, PA USA

**Keywords:** Oral microbiome, Oral microbiota, Salivary microbiome, Lifestyle, Nepali populations

## Abstract

**Background:**

Lifestyle plays an important role in shaping the gut microbiome. However, its contributions to the oral microbiome remain less clear, due to the confounding effects of geography and methodology in investigations of populations studied to date. Furthermore, while the oral microbiome seems to differ between foraging and industrialized populations, we lack insight into whether transitions to and away from agrarian lifestyles shape the oral microbiota. Given the growing interest in so-called “vanishing microbiomes” potentially being a risk factor for increased disease prevalence in industrialized populations, it is important that we distinguish lifestyle from geography in the study of microbiomes across populations.

**Results:**

Here, we investigate salivary microbiomes of 63 Nepali individuals representing a spectrum of lifestyles: foraging, subsistence farming (individuals that transitioned from foraging to farming within the last 50 years), agriculturalists (individuals that have transitioned to farming for at least 300 years), and industrialists (expatriates that immigrated to the USA within the last 20 years). We characterize the role of lifestyle in microbial diversity, identify microbes that differ between lifestyles, and pinpoint specific lifestyle factors that may be contributing to differences in the microbiomes across populations. Contrary to prevailing views, when geography is controlled for, oral microbiome alpha diversity does not differ significantly across lifestyles. Microbiome composition, however, follows the gradient of lifestyles from foraging through agrarianism to industrialism, supporting the notion that lifestyle indeed plays a role in the oral microbiome. Relative abundances of several individual taxa, including *Streptobacillus* and an unclassified Porphyromonadaceae genus, also mirror lifestyle. Finally, we identify specific lifestyle factors associated with microbiome composition across the gradient of lifestyles, including smoking and grain sources.

**Conclusion:**

Our findings demonstrate that by studying populations within Nepal, we can isolate an important role of lifestyle in determining oral microbiome composition. In doing so, we highlight the potential contributions of several lifestyle factors, underlining the importance of carefully examining the oral microbiome across lifestyles to improve our understanding of global microbiomes.

Video Abstract

**Supplementary Information:**

The online version contains supplementary material available at 10.1186/s40168-024-01941-7.

## अध्ययन संक्षेप (Abstract in Nepali)

**सारांश पृष्ठभूमि:**

मानवको आन्द्रामा ब्याक्टेरिया, भाइरस, फंगस (ढुसी), लगायत अरू विभिन्न प्रकारका सूक्ष्मजीवहरूका समुदाय पाईन्छन्, जसलाई माइक्रोबायोम भनिन्छ। आन्द्रामा हुने माइक्रोबायोमले मानवको पाचन, प्रतिरक्षा प्रणाली, र अन्य शारीरिक प्रक्रियाहरूमा योगदान पुर्याउने हुनाले यिनले मानव स्वास्थ्य र रोगको निर्धारण गर्नमा महत्त्वपूर्ण भूमिका खेल्छन्। त्यसैगरी मानव मुखमा पनि सूक्ष्मजीवहरूका समुदायहरू हुन्छन् जसले दाँत र गिजालाई स्वस्थ राख्नमा योगदान पुर्‍याउँछन्। जीवनशैलीले आन्द्राको माइक्रोबायोमलाई आकार दिन महत्त्वपूर्ण भूमिका खेल्छ। साथै, जङलमा निर्भर भएर प्रकृतिक जीवनशैली जिउने र औध्योगिक्रित भएका मानव समुदायहरुको बीच मुखको माइक्रोबायोममा धेरै भिन्नता देखिन्छ। जङलमा निर्भर भएर प्रकृतिक जीवनशैली जिउने मानव समुदायहरुमा पाईने धेरै सूक्ष्मजीवहरू औद्योगिक जनसमुदायहरुमा मा पाईन छाडिसकेका छन् र यस्ता ‘विलुप्त माइक्रोबायोम’ बिभिन्न रोगहरुका जोखिमका कारक हुन सक्ने हुँदा चासोका बिषय बन्न पुगेका छन्। तथापि, अहिलेसम्मका अनुसन्धानमा जीवनशैलिको परिवर्तन सँगै मुखको माइक्रोबायोममा के कस्ता परिवर्तनहरु आउँछन् भन्ने कुरा स्पष्ट हुन सकेको छैन। विभिन्न जीवनशैली ब्यथीत गर्ने मानिसहरु बीच जीवनशैलीको साथसाथै भौगोलिक भिन्नता पनि हुने गर्दछ। तसर्थ, मनिसहरुका मुखका माईक्रोबायोममा जीवनशैली र भूगोलको असरलाई छुट्याउनु महत्त्वपूर्ण देखिएको छ।

**परिणामहरू:**

यहाँ, हामीले ६३ जना नेपाली व्यक्तिको थुक संकलन गरेर मुखका माइक्रोबायोमको अनुसन्धान गर्यौं। हाम्रो अध्ययनमा संलग्न भएका ब्यक्तिहरुले विभिन्न जीवनशैलीको प्रतिनिधित्व गर्छन्: जङलमा निर्भर भएर प्रकृतिक जीवनशैली जिउने, जङलबाट कृषितर्फ हालसालै परिवर्तन भएका (अन्तिम ५० वर्ष भित्र), कृषक (बिगतका ३०० वर्ष भन्दा बढी समयदेखि कृषिमा संलग्न भएका), र औद्योगिक (अन्तिम २० वर्ष भित्र संयुक्त राज्य अमेरिका मा आप्रवासन गरेका नेपालीहरु)। हामीले यस् अध्ययनमा जीवनशैलीले मुखका माईक्रोबायोमको विविधतामा कसरी योगदान पुर्याउँछन् भन्ने कुरको विश्लेषण गरेका छौं, जीवनशैलीहरू बीच फरक पर्ने सूक्ष्मजीवहरूको पहिचान गरेका छौं, र माइक्रोबायोममा फरक पार्ने कारकहरूको पहिचान गरेका छौं।

प्रचलित दृष्टिकोणहरू विपरीत, भूगोललाई नियन्त्रण गर्दा, बिभिन्न जीवनशैलीहरू बीच मुखको माइक्रोबायोममा हुने सूक्ष्मजीवहरूको सन्ख्या (अल्फा विविधता) मा धेरै फरक देखिएन, तर मुखको माइक्रोबायोमको संरचना भने जीवनशैलीको क्रमिक चरणसँग मिल्दोजुल्दो देखियो, जसले देखाउँछ कि जीवनशैलीले मुखको माइक्रोबायोममा को संरचना निर्धारण गर्न एउटा प्रमुख भूमिका खेल्दछ। स्ट्रेप्टोबासिलस र असम्प्रेषित पोरफिरोमोनाडासिए जीनस सहितका केही सूक्ष्मजीवहरूको सापेक्ष सापेक्षिक प्रचुरता पनि जीवनशैलीसँग मेल खाने हामीले यस् अध्ययनबाट थाहा पाएका छौं। अन्ततः, हामीले जीवनशैलीको क्रमिक चरणमा माइक्रोबायोम संरचनासँग सम्बन्धित विशिष्ट जीवनशैलीसँग आबद्ध हुने धूम्रपान र अनाज जस्ता कारकहरूको पहिचान गरेका छौं।

**निष्कर्ष:**

हाम्रा निष्कर्षहरूले प्रष्ट परेका छन कि भूगोललाई नियन्त्रण गर्दा, हाम्रो जीवनशैलीले हाम्रा मुखको माइक्रोबायोमको संरचना निर्धारण गर्ने महत्त्वपूर्ण भूमिका खेल्दो रहेछ। यस् अध्ययनले मानव समुदायहरुमा मुखको माइक्रोबायोमको अध्ययन गरेमा माइक्रोबायोमबारेको हाम्रो बुझाइमा सुधार ल्याउन सकिने पनि देखाउँछ। बढ्दो शहरीकरण सँगसँगै नेपालीहरुको माइक्रोबायोममा परिवर्तन हुँदै गरेको र यस्ता परिवर्तनहरुले नेपालीहरुमा बिभिन्न दीर्घकालीन रोगहरुको व्यापकता बढाउन सक्ने हुनाले यस् सम्बन्धमा उपर्योक्त निकायहरुको ध्यानाकर्षण हुनुपर्ने आवश्यकता पनि यस् अध्ययनले प्रष्ट पारेको छ।

## Introduction

Throughout the last 300,000 years, our species experienced continual cultural transformation marked by milestones such as the development and use of tools, specialized division of labor, and urbanization [1]. These cultural shifts profoundly influenced both human societies and biology. One major transition in recent human history was the shift in subsistence strategy from hunting and gathering to agriculture and subsequently to industrialization. Such transitions encompass multifaceted lifestyle changes, including shifts in diet, population density, infectious disease burden, habitat, and other environmental factors [2]. These factors individually play pivotal roles in shaping the human microbiome—the diverse collection of bacteria, archaea, fungi, and other eukaryotes, and viruses that inhabit our bodies [3–6].

Understanding the role of subsistence strategy and accompanying lifestyle transitions has become a major focus of microbiome research [7–10]. Numerous studies show that the gut microbiome shifts with industrialization. Specifically, industrialized populations generally exhibit lower gut microbiome alpha diversity compared with traditional populations, often lacking microbes commonly found in traditional populations, such as *Prevotella* and *Treponema* [5, 11–13]. These differences are associated with diet, drinking water source, and social structure [13–16]. While considerable progress has been made in understanding how the gut microbiome differs across lifestyles, differences in the oral microbiome across these transitions remain largely uncharacterized. Addressing this gap is crucial considering both the role of the oral microbiome in oral and systemic health [17–19] and also its prominence in the context of ancient DNA research [10, 20]. Therefore, it is important to expand studies of the oral microbiome to encompass diverse global populations practicing different subsistence strategies.

Much of our understanding of the oral microbiome across subsistence strategies comes from a limited number of studies exploring the oral microbiomes of non-industrialized populations derived from ancient dental calculus [20, 21], although there have been increasing efforts to investigate that of modern-day populations as well [22–27]. Studies of modern-day global populations report that oral microbial diversity decreases with industrialization, with microbiome composition exhibiting differences based on lifestyle as well. More specifically, relative abundances of *Neisseria*, *Haemophilus*,* Prevotella*, and *Streptococcus* are lower in industrialized populations compared with traditional populations from the remote regions of the Philippines, Venezuela, Uganda, Malaysia, and South Africa [23, 24, 26, 28, 29]. However, several potential confounding factors may underlie the reported differences.

First, geography is associated with the microbiome [13, 30–32]. Distinguishing between the potential effects of geography versus lifestyle remains challenging, given the often large geographic variability between populations practicing different lifestyles. More specifically, samples from industrialized populations are generally collected from populations in the USA or Europe, whereas traditional populations might be collected at entirely different latitudes or continents [33]. As a result, it has been difficult to separate the effect of lifestyle and geography.

Second, some studies incorporate publicly available microbiome data from industrialized individuals with newly generated sequencing data from traditional populations [22, 24]. Technical discrepancies in sample collection, processing, and sequencing can influence microbiome study outcomes, raising questions about whether observed variations are due to lifestyle or technical factors [34, 35].

Third, studies that effectively control for technical effects are primarily focused on traditional populations [26, 36]. As a result, we lack an understanding of how the oral microbiome differs across the entire spectrum of lifestyles, from traditional to industrialized, while adequately controlling for temporal, geographical, and technical variation.

Finally, many existing studies make comparisons between distinct lifestyles, comparing traditional foragers/hunters and gatherers with agriculturalists and completely industrialized populations, without evaluating the subtle nature of microbiome shifts to and away from agriculturalist lifestyles [24, 27]. To our knowledge, there is no investigation that has comprehensively examined the entire lifestyle gradient—from hunting and gathering, to subsistence farming, to commercial farming, to early industrialization, and to established industrialization. It is important that we also examine these types of lifestyle transitions in modern-day settings, as they have been demonstrated to play major roles in shaping the oral microbiome throughout human history via analyses of ancient dental calculus. For example, increasing levels of putatively pathogenic microbes in the oral microbiome are attributed to the Neolithic Revolution and increased starch consumption with the rise of agricultural practices [37, 38]. Whether this pattern holds in modern-day lifestyle transitions remains unclear.

To address these gaps, we characterized the salivary microbiota of Nepali individuals across a spectrum of human subsistence strategies, from traditional foragers to agriculturalists. Importantly, we are able to isolate the role of lifestyle from confounders, as geographical and technical variability are controlled for in the study with the inclusion of multiple lifestyles solely from Nepal. We specifically investigated six ethnically Nepali populations across four lifestyles. The Chepang represent foragers, the Raji and Raute are hunters and gatherers that recently settled and began subsistence farming in the 1980s, the Tharu and Newars living in Nepal are agriculturalists, and the Nepali expatriates living in the USA represent a population that recently transitioned to industrialization. We also include Americans of European descent as representatives of a fully industrialized population, for a total of five lifestyles. We demonstrate that oral microbiome composition differs along a gradient of traditional to industrialized lifestyles but, unlike that of the gut microbiome, differences are relatively subtle. By integrating questionnaire-based data encompassing diverse lifestyle variables such as diet, education, and medical practices, we identify specific lifestyle factors associated with oral microbial compositional changes. Finally, we examine the gut-oral microbiome axis to evaluate whether the degree of intra-individual similarity between the two sites differs across lifestyles. These results demonstrate that like the gut microbiome, the oral microbiome mirrors lifestyle.

## Results

### Description of populations

**Fig. 1:**
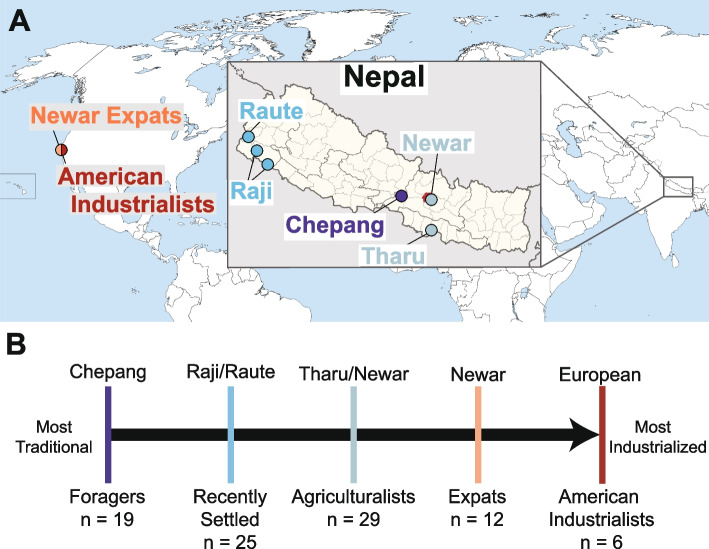
Sampling locations of all Nepal- and USA-based populations. **A** Locations of the populations sampled in Nepal and the USA. The US populations are specifically from the Northern California region. Location of Kathmandu is indicated in red on the Nepal map. Colors correspond to lifestyle groupings as described in **B**. **B** Oral microbiome samples were collected from individuals that span a spectrum of lifestyles, from nomadic foraging populations (dark blue), to populations that recently transitioned from foraging to small scale agriculture (teal), to established small scale agriculturalists (sky blue), to Nepali expats residing in the US practicing an industrialized lifestyle (peach), and to American industrialists (red). Sample sizes for each lifestyle category are indicated

We investigated the oral microbiome in diverse Nepali populations practicing a spectrum of lifestyles and an American population representing an industrialized lifestyle. In total, we examined individuals practicing five lifestyles: foragers, recently settled, agriculturalists, expats, and American industrialists. These lifestyles were defined a priori by subsistence strategy. However, a number of specific lifestyle factors differ across groups in addition to subsistence strategies, such as household size, diet, and smoking habits. Briefly, the Nepali individuals in this study belonged to five ethnic groups native to Nepal–Chepang (*n* = 18), Raji (*n* = 11), Raute (*n* = 14), Tharu (*n* = 20), and Newar (*n* = 8) (Fig. 1, Table S1). We also included expatriate Newar (*n* = 12) and European-Americans (*n* = 6), both of whom reside in the San Francisco Bay Area. The Chepang, numbering 84,400 [39], are foragers who primarily reside in small communities of remote, isolated villages within the hills of the lower Himalaya in central Nepal. The Chepang village in this study lacks modern amenities such as electricity, running water, and other indicators of urbanization. While they supplement their diets with food grown via slash-and-burn agriculture, the low productivity of the hilly terrain compels them to heavily depend on foraged jungle foods such as undomesticated tubers and wild nettle (*sisnu*). The Raji and Raute, previously nomadic foragers, transitioned to subsistence agriculture within the past 50 years. The Raute and Raji are among the smallest ethnic groups in Nepal. The Raute, with roughly 550 individuals [39], reside in the far western hills of Nepal, whereas the Raji, numbering 5100 [39], inhabit the neighboring Terai plains. These two populations will be referred to as “recently settled” in this study, due to the transitional nature of their lifestyle. By contrast, the Tharu and Newar, two of the largest ethnic groups in Nepal numbering 1.8 and 1.3 million, respectively [39], practice agriculture. The Tharu, hailing from the Terai plains in Southern Nepal, fully transitioned to agriculture about 300 years ago. The Newar originate from Kathmandu valley and are renowned for their cultural and economic contributions to Nepal. Although increasing urbanization of Kathmandu valley has afforded some Newar individuals access to industrialized comforts, the population in this study resides in a relatively rural village on the outskirts of Kathmandu valley and primarily engages in agriculture. For those reasons, in this study, both the Tharu and Newar will be referred to as the established agriculturalists. The expatriate Newar in the USA (“expats”) also originated from the Kathmandu valley and emigrated within the past 20 years, settling in the USA in their mid-30s. The Chepang, Raji, Raute, and Tharu individuals sampled here largely overlap with a previous study of Nepali gut microbiomes [15]. Both the fecal samples in this previous study and the saliva samples in this current study were collected concurrently. By focusing on individuals across a range of lifestyles within a confined geographic region, our study aims to discern oral microbial signatures of lifestyle without the confounding effects of geography, climate, and technical factors.

### Lifestyle factors differ across populations

Given that multiple dietary, environmental, and socio-economic factors differ across populations in Nepal, we administered a survey to capture the specific lifestyle factors that differ across the Nepali populations (Table S1). We used Random Forests to evaluate the ability of the 37 lifestyle factors obtained from the survey questions to classify individuals according to their respective lifestyle groups. Nepali individuals are correctly assigned to their respective lifestyle categories with 92.06% overall accuracy (Fig. S1), suggesting that the populations in our study are highly distinguishable based on these factors.

We then performed correspondence analysis (CA) to determine which specific variables distinguish the lifestyles. Our analysis reveals a significant dependence between the samples and lifestyle factors (*p* = 0.004; chi-square test of independence). Individuals cluster closely in the top two dimensions of CA based on their lifestyle group, as do similar specific lifestyle variables (i.e., education and literacy, Fig. S2 A). Specifically, CA axis 1 follows the lifestyle trend (*p* = 3.26 × 10^−14^; Jonckheere-Terpstra test), whereas CA axis 2 does not (*p* = 0.21; Jonckheere-Terpstra test, Fig. S2 B-C). The top 10 contributing lifestyle factors to CA1 include *sisnu* consumption, fuel source, and literacy (Fig. S2 D). For example, the Chepang consume *sisnu* most frequently, use solid fuel for cooking, and have low literacy rates, while the Expats do not consume *sisnu*, use gas or electricity for cooking, and are literate. By contrast, the top contributing factors to CA2 include behavioral factors like smoking and alcohol consumption (Fig. S2 E). The high predictive capabilities of the Random Forest analysis and discrimination between lifestyle categories based on specific lifestyle factors suggest that the lifestyles are both well-defined by subsistence strategy and effectively described by the survey metadata.

### Oral microbiome diversity does not differ across Nepali populations

Fecal samples concurrently collected with the saliva from the Chepang, Raji, Raute, and Tharu individuals previously revealed pronounced gut microbiome compositional differences across the gradient of lifestyles from foraging to industrialized [15]. To evaluate whether oral microbiome compositional differences align with the continuum of lifestyles, we initially characterized the oral microbiome via saliva samples collected from 89 individuals across five lifestyles (Table S1). Recognizing the potential for DNA extraction methodology to introduce variability in microbiome studies [34, 35], we performed DNA extraction using two different kits, Qiagen QIAamp MinElute Virus Spin kit and MO BIO PowerSoil DNA, to ensure the robustness of our conclusions. Overall microbiome composition and diversity are consistent between the two kits (Fig. S3). Consequently, all subsequent analyses were performed using the data obtained from the Qiagen kit for simplicity, which resulted in 69 individuals that passed quality control steps (see the “Methods” section).

**Fig. 2:**
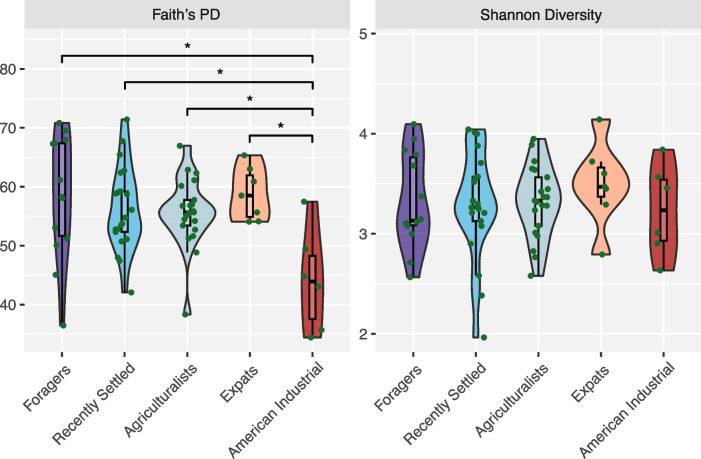
Alpha diversity does not significantly differ by lifestyle in Nepal. Faith’s phylogenetic diversity (Faith’s PD—left) and Shannon alpha diversity (Shannon Diversity—right) shown for all individuals, grouped by lifestyle. Lifestyles are ordered from most traditional (foragers) to most industrialized (American industrialists), left to right. No significant difference detected across lifestyles for Shannon alpha diversity (*p* = 0.8, Kruskal–Wallis), but a marginally significant difference detected for Faith’s phylogenetic diversity (*p* = 0.028, Kruskal–Wallis). Notably, those significant differences only occur between the American industrialists and other lifestyle groups, not between Nepali individuals residing in Nepal or the USA. Significant differences (*p* < 0.05) between specific populations are indicated (*)

We first evaluated whether overall microbiome diversity differed across lifestyle groups, as decreasing diversity is typically thought of as a hallmark of traditional to industrialized lifestyle transitions [12, 24, 26]. We observe no significant difference in Shannon diversity across the lifestyle groups (*p* > 0.05, Kruskal–Wallis; Fig. 2), but there is a significant difference in Faith’s phylogenetic diversity between the lifestyles (*p* = 0.028; Kruskal–Wallis, Fig. 2). A post hoc pairwise comparison demonstrates that the American industrialists are driving the differences in Faith’s phylogenetic diversity (American Industrialists vs. other lifestyles: *p* < 0.05, Dunn’s post hoc test). Notably, there is no significant difference in Faith’s phylogenetic diversity between the four Nepali populations (*p* > 0.05; Kruskal–Wallis). These findings remain largely consistent with other alpha diversity metrics across both extraction kits (see the “Methods” section, Fig. S4–S5). Aligning with the observations in the gut microbiomes of these individuals [15], our results indicate that oral microbiome diversity does not correlate with lifestyle differences within Nepal when geography is controlled for. Notably, our sample sizes, while modest, are larger than most other oral microbiome studies examining traditional lifestyles [22–24, 26], underscoring that the lack of signal is not due to insufficient power. It is also important to note that the difference in microbiome diversity is observed using Faith’s phylogenetic diversity and not using Shannon’s diversity. While both within-sample diversity metrics, Shannon’s diversity accounts for species abundance and evenness without consideration of underlying phylogenetic structure, whereas Faith’s phylogenetic diversity accounts for the total phylogenetic breadth of the residing taxa. As a result, our results suggest that while the microbes residing in the Nepali individuals cover a larger extent of the phylogenetic tree than the American industrialists, the relative evenness is fairly equal between the populations.

### Oral microbiome composition differs across lifestyles

**Fig. 3:**
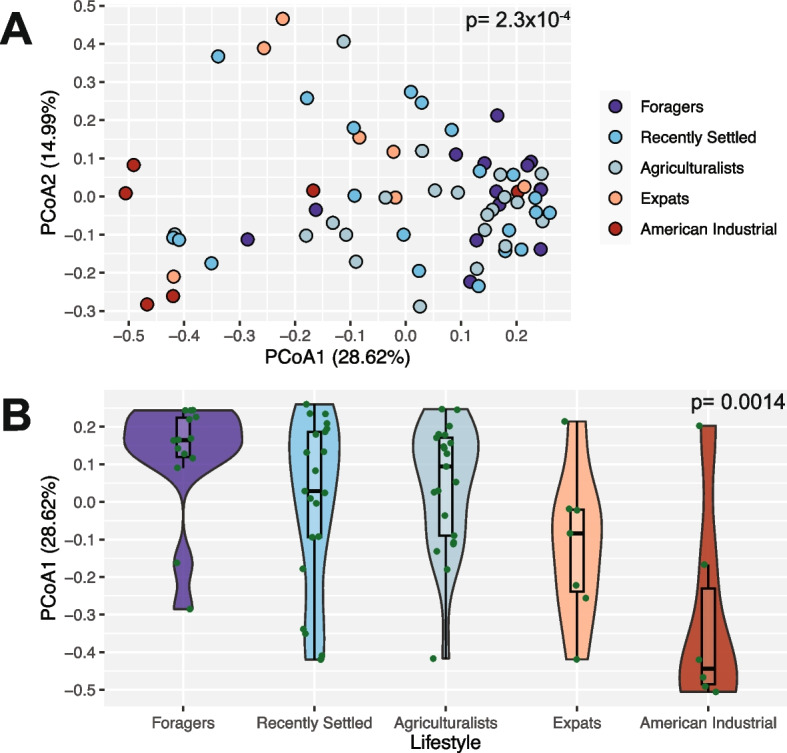
Oral microbiome composition significantly differs based on lifestyle.** A** Microbiome composition varies significantly with lifestyle (*p* = 2.3 × 10^−4^, PERMANOVA). The PCoA plot shows individuals ordinated based on Bray–Curtis distance and colored by lifestyle. **B** The distribution of individuals along PCoA axis 1 follows the lifestyle gradient, from traditional to industrial (*p* = 0.0014, Jonckheere-Terpstra test). Lifestyles are ordered from most traditional (foragers) to most industrialized (American industrialists), left to right

Unlike microbiome diversity, microbiome composition varies across lifestyles. We calculated between-sample Bray–Curtis distances to measure beta diversity [146], revealing that oral microbiome composition varies significantly with lifestyle (*p* = 2.3 × 10^−4^; PERMANOVA, Fig. 3A). This relationship remains significant even when accounting for sex across all individuals and both sex and age across the Nepali individuals (*p* = 1.5 × 10^−4^, *p* = 0.025; respectively, PERMANOVA). Subsequent pairwise comparisons demonstrate multiple significant differences between specific pairs of groups (foragers vs. American industrialists, foragers vs. expats, agriculturalists vs. American industrialists: *p* < 0.05, pairwise PERMANOVA). When visualized via principal coordinate analysis (PCoA), the first axis (PCoA1, explaining 28.62% of microbiome variation) follows the lifestyle gradient (*p* = 0.0014; Jonckheere-Terpstra test, Fig. 3B), with the expatriates and Americans being most different from the traditional Nepali populations. This pattern is consistent across data from both extraction kits (Fig. S6 B–D) and with UniFrac metrics (Figs. S7–S8) [40]. These compositional differences across groups, however, are fairly subtle, with the classification of microbiomes into lifestyle groupings via Random Forests not being better than expected by random chance (43.48% accuracy, Fig. S9).

**Fig. 4:**
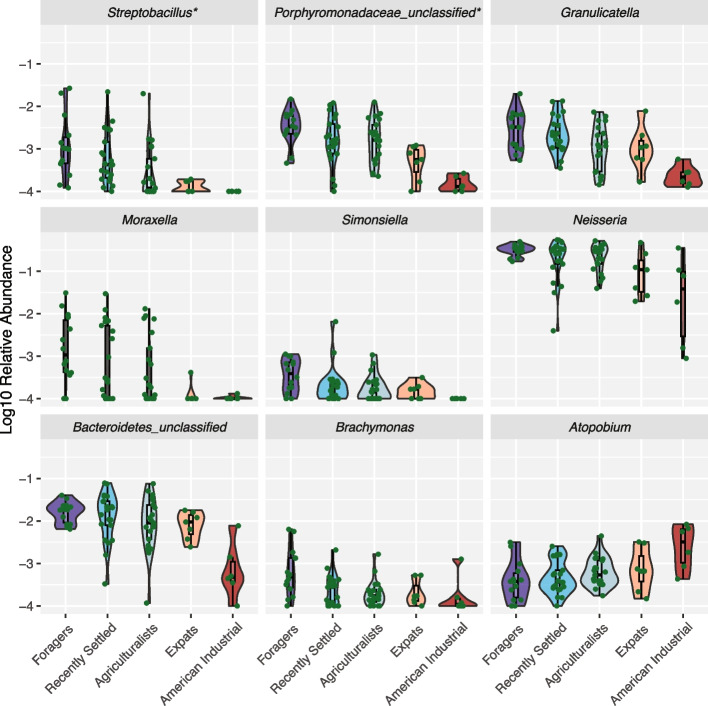
Abundances of nine genera significantly follow the lifestyle gradient. The relative abundances of nine genera significantly follow the lifestyle gradient via a Jonckheere-Terpstra test followed by Benjamini–Hochberg correction (adjusted *p* < 0.05). Lifestyles are ordered from most traditional (foragers) to most industrialized (American industrialists), left to right. All taxa have been log10-transformed for visualization purposes. Taxa marked with * are also significantly differentially abundant across lifestyles based on ALDEx2. Most taxa tend to decrease in relative abundance as the lifestyles transition from more traditional to industrial, while the abundance of *Atopobium* increases. ﻿

Because overall microbiome composition differed based on lifestyle, we next determined which specific taxa differed across the lifestyles. To do this, we conducted differential abundance analysis using ALDEx2, which accounts for compositionality in its application [41]. We observe that 2 of the 111 oral genera (1.8%) were significantly differentially abundant after accounting for multiple tests, namely *Streptobacillus* and an unclassified Porphyromonadaceae genus (padj = 0.011 and 0.021, respectively; Kruskal–Wallis) (Table S2). These results are robust to the inclusion of sex and age as covariates (Table S3). We also implemented an alternative approach for identifying which taxa were following the lifestyle gradient by performing the Jonckheere-Terpstra test for all genera and correcting for multiple tests. We find that nine genera significantly followed the lifestyle gradient, including the two that were also identified using ALDEx2 (Fig. 4, Table S4). Eight genera—*Streptobacillus*, *Porphyromonadaceae_unclassified*,* Granulicatella*,* Moraxella*,* Simonsiella*,* Neisseria*,* Bacteroidetes_unclassified*, and* Brachymonas—*show decreasing abundance with industrialization, consistent with the trend observed in Faith’s phylogenetic diversity. The only exception is *Atopobium*, which shows the opposite trend of increasing abundance with industrialization (Fig. 4). To identify whether these trends were driven primarily by the industrialists, we analyzed the lifestyle gradient for only the Nepali groups. We find that only *Streptobacillus* significantly follows the lifestyle gradient within Nepali individuals after correcting for multiple tests. Interestingly, when applying the same approach to all individuals except the foragers, we also find that, again, only *Streptobacillus* significantly follows the lifestyle gradient. These results confirm that the extreme ends of the lifestyle gradient have substantially different microbial relative abundances, with the intermediate stages exhibiting more subtle differences in relative abundance.

### Grain type is associated with microbiome differences across lifestyles

Given that overall microbiome composition mirrored the transition of lifestyles within Nepal, we sought to identify lifestyle factors that potentially underlie these differences. To do this, we first started at a broad scale by comparing the major axes defining oral microbiome composition and the lifestyle variables from PCoA and CA, respectively. We calculated the correlation between the first three CA axes, which cumulatively captured 37.14% of the variation in the lifestyle survey data, and the first three PCoA axes, which cumulatively captured 49.35% of the variation in the microbiome data. We observe a significant correlation between PCoA2 and CA2, which is primarily comprised of behavioral lifestyle factors like tobacco and alcohol use and distinguishes the recently settled populations from foragers and agriculturalists (*p* = 0.03; rho =  − 0.27; Spearman correlation, Fig. S10 A). No significant correlation is observed between the CA axes and alpha diversity (Fig. S10 B).

**Fig. 5:**
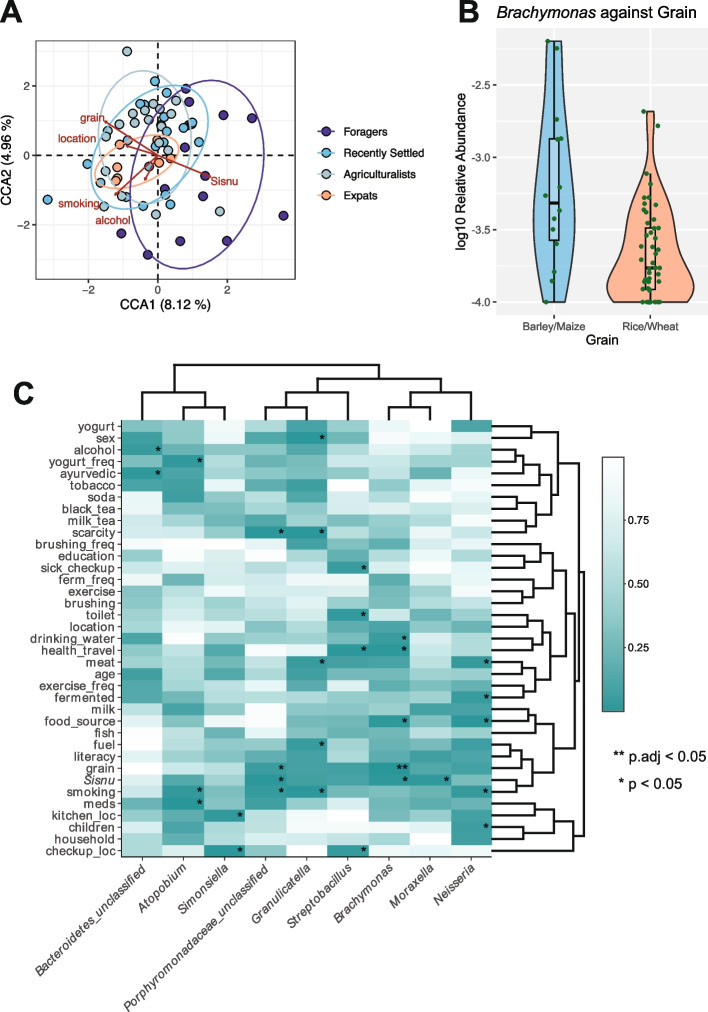
Alcohol, smoking, location, *sisnu*, and grain type are associated with the oral microbiome. **A** There are significant associations between lifestyle factors and the microbiome as observed via CCA. Points represent individuals and color represents corresponding lifestyle, with ellipses around each population. Red arrows represent the lifestyle factors significantly associated with the microbiome. A total of 15 lifestyle factors were inputted into the CCA model based on contribution to each CA axis. **B** *Brachymonas* is significantly associated with grain type consumed (padj = 0.022). Specifically, relative abundance is higher in individuals who report barley and maize consumption compared with rice and wheat. Taxa were log10 transformed for visualization. **C** Several specific lifestyle factors are associated with individual oral genera. Significant associations based on a nominal p-value threshold are indicated with *. The significant association based on an adjusted *p*-value threshold is indicated with **

We then identified which specific lifestyle factors are associated with the observed differences in microbiome composition. As testing all 37 measured lifestyle factors would be prohibitive due to multicollinearity, we selected the top 15 key lifestyle distinguishing factors based on their contributions to the first two CA axes (Fig. S2). We used these factors to perform canonical correspondence analysis (CCA) to determine which variables are associated with shifts in the microbiome. We find significant associations between these lifestyle distinguishing factors and the oral microbiome composition among the Nepalis (*p* = 0.013; ANOVA); with the top factors being alcohol consumption, smoking habits, location, *sisnu* consumption, and grain type (*p* = 0.044, 0.001, 0.003, 0.003, and 0.027, respectively, ANOVA) (Fig. 5A).

Finally, we identified associations between the nine taxa that are differentially abundant across lifestyle groupings (Table S4) and the 37 lifestyle factors included in our survey via linear models. Out of the 333 associations tested, we find that *Brachymonas* is significantly associated with grain consumption at an adjusted *p*-value, with a higher abundance of this taxon observed in individuals who primarily consume barley and maize compared with those who primarily consume rice and wheat (padj = 0.022; Fig. 5B). Furthermore, we observe an additional 27 associations that are significant at a nominal *p*-value < 0.05 (Fig. 5C, Table S5). For example, we observe that the relative abundances of *Granulicatella*, *Neisseria*, and *Porphyromonadaceae_unclassified* are higher in non-smokers, whereas *Atopobium* relative abundance is lower in non-smokers (*p* = 0.006, *p* = 0.032, *p* = 0.033, and *p* = 0.023, respectively, Fig. S11 A). Similarly, the relative abundances of *Brachymonas*,* Moraxella*, and *Porphyromonadaceae_unclassified* are higher in individuals that consume *sisnu* (*p* = 0.014, *p* = 0.019, and *p* = 0.012, respectively, Fig. S11 B). Notably, while both smoking and alcohol are associated with the oral microbiome and both factors are top contributors to lifestyle, neither of these factors significantly follow the lifestyle gradient from traditional to expatriate (*p* > 0.05; Cochran-Armitage test, Fig. S12). These results demonstrate that a variety of lifestyle factors potentially underlie the differences in oral microbiome composition observed between lifestyles within Nepal.

### Predicted metabolism pathways are differentially abundant across lifestyles

In addition to the taxonomic differences observed between lifestyle groups, the predicted functional potential of the microbiome significantly differs as well. Based on the use of PICRUSt2 [42], predicted functional abundance significantly varies with lifestyle (*p* = 0.0036; PERMANOVA, Fig. S13 A). The top two PCA axes both significantly follow the lifestyle gradient (PC1: *p* = 0.049, PC2: *p* = 0.0064; Jonckheere-Terpstra test, Fig. S13 B). We note that while the taxa present in Nepali oral microbiomes seem to be fairly closely represented in the reference taxonomy used to predict gene content (average NSTI = 0.037), there are many caveats to predictive methods such as PICRUSt2 and these results should be viewed as hypothesis-generating [43].

To identify specific potential functional differences across lifestyles, we conducted differential abundance testing with ALDEx2 using the predicted abundances of 109 pathways. Although none are significant after multiple test corrections, 22 pathways are significant at a nominal *p*-value of *p* < 0.05 (Table S6), 13 of which are classified as metabolism pathways (Fig. S14A). These metabolism pathways can be categorized into 7 classes, some of which increase in abundance with increasing industrialization, including lipid metabolism and glycan biosynthesis, while others decrease, such as xenobiotics degradation and microbial metabolism in diverse environments (Fig. S14B). General transporter proteins (ATP-binding cassette transporters—padj = 0.0013, phosphotransferase system—padj = 0.0013) and degradation pathways (aminobenzoate degradation—padj = 0.0028) are significantly enriched via enrichment analysis (Fig. S15). Finally, the top 10 most significant pathways from ALDEx2 were further examined to identify the top contributing microbes and whether they differ by lifestyle. *Fusobacterium* is one of the top taxa contributing to platinum resistance (Fig. S16 A). There is a significant enrichment of *Fusobacterium* in the traditional Nepali populations compared with industrialized populations (*p* = 0.0037; Kruskal–Wallis test, Fig. S16 B). Overall, predicted metabolism pathways significantly differ across lifestyles, mirroring the taxonomic gradient across lifestyles in Nepal.

### Microbial network structure varies across lifestyles

**Fig. 6:**
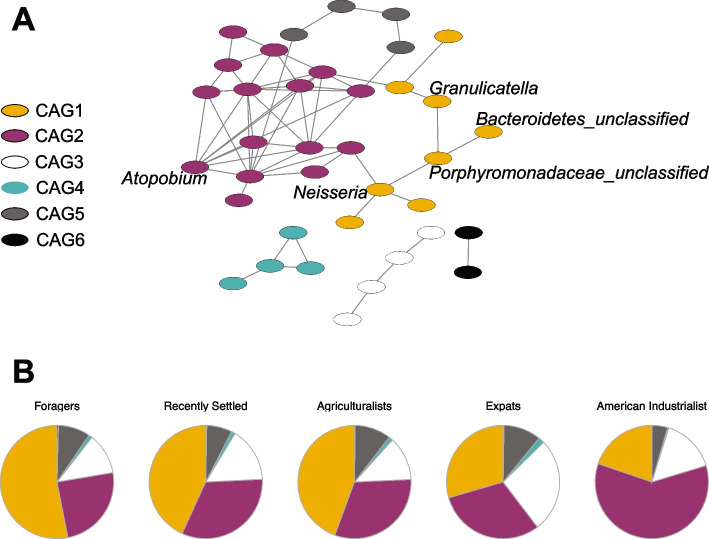
Differentially abundant taxa are highly connected in the oral microbiome co-occurrence network. **A** The SparCC module in the SpiecEasi package was used to generate a network from 111 genera. Network of 37 nodes with at least one significant edge is shown, with 6 co-abundance groups (CAGs) indicated by node color. Labeled nodes indicate genera that were identified as significantly differentially abundant across lifestyles. **B** Proportions of CAGs vary across lifestyle. Specifically, CAG1 decreases with industrialization, whereas CAG2 increases with industrialization

We then investigated network structure to determine whether community structure differs across lifestyles. We used the SparCC module in the SpiecEasi package [44, 45] to generate a network from all 111 genera observed in this study. The resulting network consists of 37 nodes with at least one edge and 6 co-abundance groups, with a modularity of 0.45 (Fig. 6A). Among the taxa identified as following the lifestyle gradient, 5 out of the 9 are connected to at least one other taxon, with *Porphyromonadaceae_unclassified*,* Neisseria*,* Bacteroidetes_unclassified*, and *Granulicatella* being in the same co-abundance group (CAG1), whereas *Atopobium* is in a separate co-abundance group (CAG2). Interestingly, the proportions of CAGs differ across lifestyles, with CAG1 decreasing with industrialization and CAG2 increasing with industrialization (Fig. 6B). These results demonstrate that community network structure differs along the lifestyle gradient, in addition to individual microbial taxa and predicted functional potential.

### Oral-gut microbiome distance decreases with agrarianism

**Fig. 7:**
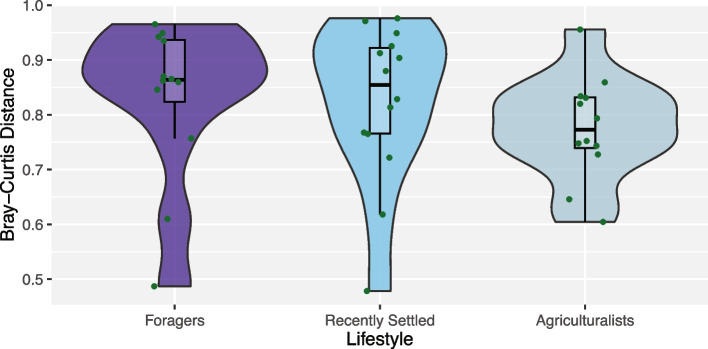
Correlation between the oral and gut microbiomes within an individual strengthens with agrarianism. Microbiome dissimilarity (as measured by Bray–Curtis dissimilarity) between the gut and oral microbiomes within an individual decreases across the gradient of lifestyles from traditional to agrarian for individuals residing in Nepal, albeit not significantly (*p* = 0.11; Jonckheere-Terpstra test)

Finally, we examined the role of lifestyle along the oral-gut microbiome axis. While different lifestyle factors independently associated with the oral and gut microbiomes, the compositional similarity between the two sites within an individual increases with the extent of urbanization [25]. Thus, we were interested in assessing whether there was a similar association across lifestyles, in which we might expect to see increasing intra-individual similarity across the gradient of lifestyles from traditional to agrarian. To do this, we examined individuals for whom both oral and gut microbiome data were collected concurrently, for a total of 12 foragers, 14 recently settled individuals, and 12 agriculturalists (Table S7), and calculated Bray–Curtis dissimilarity between the oral and gut microbiomes. We find that intra-individual oral-gut microbiome dissimilarity decreases, and therefore similarity increases, across the gradient of traditional to agrarian lifestyle as predicted, although this trend is not statistically significant (*p* = 0.11; Jonckheere-Terpstra test, Fig. 7). We, however, do observe significant similarities in composition between the two body sites across individuals (*p* = 0.013, rho =  − 0.4; Spearman correlation, Fig. S17), suggesting that we are perhaps underpowered to detect the significance of intra-individual dissimilarity at our current sample size. To verify this, we conducted a power analysis and determined that we only have 4.1% power given our measured effect size differences between groups and would require at least 62 individuals per group to detect a significant trend with 80% power (Fig. S18).

## Discussion

Much of human microbiome research is weighted towards populations living in North America and Europe, with South Asia being particularly underrepresented [46]. As a consequence, our understanding of how the oral microbiome varies across human lifestyles is extremely limited, especially with regard to non-industrialized lifestyles including foraging, hunting and gathering, and small-scale agriculturalism. Some existing studies identify differences in the oral microbiome between hunter-gatherers and farmers [24, 26]. Others investigate the role of a few specific lifestyle factors; like smoking, alcohol consumption, and diet; but only in a single, usually industrialized, population [36, 47–49]. Our study is the first comprehensive examination of the oral microbiome across a gradient of lifestyles, including transitional lifestyles—recently settled nomadic foraging individuals now practicing small-scale agriculture and expatriates transitioning from farming to industrialization—and numerous lifestyle factors characterizing each lifestyle. Importantly, we are able to isolate the role of lifestyle, as geography and technical factors are controlled for. We observe that even when controlling for geography, microbiome composition mirrors lifestyle transitions, likely due to differences in dietary and behavioral habits.

In general, populations in industrialized countries have lower gut microbiome diversity than those practicing more traditional lifestyles [5, 11–14, 16, 50, 51]. This has led to an active discussion regarding whether we should intervene to improve human health and reduce microbial loss in industrialized countries [9, 52, 53]. While it is tempting to attribute the observed diversity differences across populations to lifestyle, controlling for geography eliminates those differences [14, 54, 55]. In fact, when examining the gut microbiomes of the individuals included in this study, there was no significant difference in within-sample alpha diversity across lifestyles, even when including American industrialists [15]. Fewer studies examine oral microbiome diversity across human lifestyles, and most of them observe a decrease in diversity across lifestyles [24, 26]. That said, it is important to highlight that geography was not closely controlled for and might have confounded the results, as examining lifestyle while controlling for geography results in no difference in diversity [22].

Here, we demonstrate that alpha diversity of the oral microbiome does not significantly differ across lifestyles between Nepali individuals, aligning with findings from oral microbiome studies that control for geography [22]. Interestingly, unlike the gut microbiomes of the same individuals [15], we observe a significant decrease in Faith’s phylogenetic diversity in the American industrial population compared with Nepali individuals, including the Nepali expats. As the Nepali expats and American industrialists currently reside within the same metro area, geography is effectively controlled. There are several possible explanations for why these differences are observed between the Nepali expats and American industrialists, but not the populations within Nepal. First, the main differentiating factor may not be geography, as one might assume if only comparing the populations residing in Nepal with American industrialists, but rather lifestyle factors that have more extreme effects between industrialists and other lifestyles than between the traditional and agrarian populations within Nepal. For example, the Nepali expats included in this study tend to retain their traditional cuisine, which differs greatly from a standard American diet. Although recipes are modified to account for local ingredient availability, the main dietary components remain consistent across the ethnically Nepali populations, regardless of geography. Another possibility is that geography does drive oral microbial diversity, but can only do so during critical windows earlier in life in which oral microbiomes are malleable [56]. While gut microbiota appear to be malleable even with immigration well into adulthood [57], it is not clear if the same is true for the oral microbiome. Once a stable microbial community or host immune repertoire is established, moving to another geographic region may not result in diversity changes. The Nepali expatriates included in this study immigrated around their mid-30s, so they may have missed this window. Further investigation would be needed to tease apart these and other possible explanations for the differences in diversity we observed between Nepali individuals and American industrialists.

Similar to the gut microbiomes in the same individuals [15], oral microbiome composition mirrors lifestyle. Specifically, we observe a consistent compositional gradient when comparing individuals from traditional foraging populations (Chepang) to recently settled populations (Raji and Raute), to small-scale agriculturalists (Newar and Tharu), to immigrants (Newar) and industrialized Americans. Compared with the gut, which we reanalyzed using ALDEx2 to ensure comparability of results (Table S8), differences across lifestyles are more muted in the oral microbiome. More genera in the gut are significantly differentially abundant across lifestyles (27% in gut, 1.8% in oral), even though there was slightly more power with the oral samples due to increased sample size. This finding may be due to the increased resiliency of the salivary microbiome compared with the gut microbiome, thus resulting in fewer differences in the microbiome across lifestyles [58–62]. It is important to note that these considerations are specific to the salivary microbiome, as the microbiomes of other oral anatomical sites, including dental calculus, differ significantly from that of saliva [63–65].

When examining microbial abundance across lifestyles, most of the differentially abundant microbes decrease across the gradient of traditional to industrialized. Many of these taxa co-occur and lie in the same co-abundance group when considering the full oral microbiome network (CAG1). One such taxon is *Neisseria*, which decreases in abundance with industrialization in other lifestyle studies [24, 28]*. Neisseria* plays a beneficial role in periodontal health, possibly by preventing the colonization of pathogenic microbes [66, 67]. Its decreasing abundance aligns with the hypothesis that the loss of crucial microbes is associated with the emergence of disease in industrialized society [68]. Other previously identified lifestyle-associated oral microbes are not significantly associated with lifestyle in our study, such as *Haemophilus*,* Prevotella*, and *Streptococcus* [23, 24, 26, 29]. Instead, we observe decreasing levels of an unclassified Porphyromonadaceae genus. *Porphyromonas gingivalis* is a member of the Porphyromonadaceae family, well-established as a pathogenic oral microbe contributing to periodontal disease [69]. Further investigation is needed of this microbe in our dataset, as this would provide insight into Porphyromonadaceae as a potential oral pathogen at a global scale. *Streptobacillus* is another microbe that decreases in abundance with increasing industrialization. In North America, *Streptobacillus* is most well-known as *Streptobacillus moniliformis* for its presence in rat oral microbiomes and its role in rat bite fever [70]. By contrast, *Streptobacillus* appears as a commensal member in the human oral microbiomes of Asian populations. More specifically, *Streptobacillus hongkongensis* resides in the oral cavity of populations from Hong Kong and the United Arab Emirates [71, 72]. *Streptobacillus* has also been found in the Agta hunter-gatherers in the Philippines [73], thus suggesting that *Streptobacillus* presence in the human oral microbiome may be regionally limited to Asia. This hypothesis aligns with our study, as *Streptobacillus* is not observed in the American industrialists and is also less abundant in the expats. However, we note that our American industrialist sample size is limited, and larger sample sizes would be needed to confirm the low prevalence of this taxa in non-Asian populations. The relative abundance of differentially abundant microbes decreasing with industrialization is consistent with prior observations of decreased microbiome diversity in industrialized populations.

By contrast, the one genus that significantly increases in relative abundance with industrialization is *Atopobium.* High levels of oral *Atopobium* relative abundance are associated with a variety of negative health outcomes, many of which are more common in industrialized societies. Highly abundant in individuals with dental caries, *Atopobium* is believed to contribute to the development of dental caries as an accessory to *Streptococcus mutans*, a leading microbial cause of caries [74–76]. Oral *Atopobium* carriage is also enriched in individuals with hypertension [77], Sjögren’s Syndrome [78], and patients with severe oral mucositis, a toxicity occurring from cancer treatments [79]. Notably, we observe that *Atopobium* belongs to a separate co-abundance group from the other differentially abundant taxa, CAG2. CAG2 also contains *Veillonella*, a microbe that may be an accessory for oral pathogen colonization [80]. Future research is needed to disentangle whether *Atopobium* and other potentially pathogenic microbes play causal roles in the development of oral conditions.

Multiple specific lifestyle factors are believed to play a role in shaping the oral microbiome, such as smoking, dietary fiber, and carbohydrate sources [47, 48, 81]. Most of these factors, however, were characterized in industrialized populations and it remains unclear whether the same factors play a role in the oral microbiota of traditional populations. Using extensive survey metadata, we observe significant associations between the microbiome and 15 lifestyle factors, with smoking, grain type, and *sisnu* consumption most strongly associated.

Smoking consistently associates with oral microbiome composition across numerous industrialized populations, with increases in *Atopobium* and decreases in *Neisseria* and family-level Porphyromonadaceae being trademark indicators [48, 82–85]. We identify similar associations with smoking in our study as well. Specifically, *Atopobium* abundance is increased in smokers, whereas *Granulicatella*, *Neisseria*, and *Porphyromonadaceae_unclassified* abundances are decreased in smokers. The taxa with increased abundances in smokers cluster in the same co-abundance group, whereas *Atopobium* is found in a separate co-abundance group. Notably, while smoking prevalence rates differ across lifestyle groups, they do not follow the gradient of lifestyles from traditional to agricultural. This suggests that the association of these taxa with smoking is independent of subsistence strategy. These findings suggest that smoking habits may play an important, consistent role in defining oral microbiome community dynamics across lifestyles and highlight the importance of accounting for smoking as a factor in future studies of lifestyle and the oral microbiome.

The associations we observe between grain type and the oral microbiome in Nepali populations are of particular interest, given the proposed importance of starch-rich foods in shaping oral microbiomes along the primate lineage [86]. Carbohydrates are associated with a myriad of oral microbes, either due to their role in starch digestion or pathogenicity [87, 88]. In this study specifically, populations reported primarily consuming either barley and maize, or rice and wheat. Barley and maize differ substantially from rice and wheat in terms of phenol content, digestibility, fiber content, and glycemic index [89, 90]. More specifically, barley and maize contain higher levels of phenols, which positively associated with gut microbiome health, oral microbiome health, and overall systemic health [91, 92]. In addition, rice and wheat are digested faster than barley and maize [90]. This higher digestibility might be attributed to differences in cell structure, like thinner cell walls [93]. More starch consumption and more salivary amylase, the first step of starch digestion, may also be contributing factors. Higher salivary amylase copy number has been observed in individuals with high starch diets [94] and is associated with oral microbiome composition [95]. Furthermore, barley has higher fiber content compared with other refined grains [96], which is also associated with improved health outcomes [97]. Finally, barley is reported to have a lower glycemic index compared with rice and wheat [89], which is generally associated with more positive health outcomes [98]. We observe a significant association between grain type and *Brachymonas*, which has not been previously found to be associated with grains in human oral microbiomes. Little is known about the role of this microbe in humans, beyond its presence in healthy oral microbiomes [99–102]. Instead, this microbe has been demonstrated to decrease in abundance in the rumen microbiome of cows fed a high-grain diet [103], so further investigation is needed to fully understand this potential relationship.

We also identify a possible relationship between *sisnu* and the oral microbiome. *Sisnu*, also referred to as nettle, is a fibrous plant known for its medicinal benefits and primarily consumed by the Chepang foragers [104]. *Sisnu* consumption is a major differentiating lifestyle factor in this study, as demonstrated by correspondence analysis (Fig. S2). Although widely used in traditional medicine, little is known about its role in the microbiome. *Sisnu* shows strong antimicrobial properties against a variety of gram-positive and negative bacteria in vitro [105], although evidence is mixed [106]. Here, *Porphyromonadaceae_unclassified* relative abundance increases with *sisnu* consumption*.* Several mechanisms could explain this association. First, ingesting *sisnu* may result in lowered absolute abundance of the oral microbiome overall, with *Porphyromonadaceae_unclassified* being more resilient than other microbes. Second, the antimicrobial effects could create an expanded niche for this microbe to thrive, without impacting absolute abundance levels across the oral microbiome. Finally, the association may be unrelated to the potential antimicrobial properties of the plant, but rather nutrition. A finer-grain investigation would be needed to fully establish the underlying causes of this association, especially considering the prominence of *sisnu* as a therapeutic agent. Overall, these analyses of specific lifestyle factors associated with the oral microbiome in Nepali populations provide new insight into the role of specific dietary components and environmental factors in the oral microbiomes of non-industrialized, non-equatorial populations.

One unexpected result was the lack of an observed association with teeth brushing or flossing. Oral hygiene practices are associated with the microbiome, as the mechanical actions of brushing and flossing disrupt plaques and antimicrobial toothpastes also chemically break down biofilms. As a result, even subtle differences in types of toothpaste and brushing frequency result in changes in the plaque and salivary microbiota [107–110]. In traditional populations, miswak, also referred to as a chewing stick, is often utilized for mechanically and chemically cleaning teeth and has been demonstrated to inhibit common oral pathogens [111–115]. Finally, charcoal and ash have also been used historically for oral hygiene, although their effectiveness is highly disputed [116]. Our study included individuals who reportedly do not brush their teeth, those who use traditional methods of miswak or charcoal, and those who brush with a toothbrush and toothpaste. Unexpectedly, we observed no association with reported tooth brushing, let alone the brushing method or frequency. This may be due to undocumented variance in “brushers,” as important aspects of oral hygiene such as differing toothpaste amounts, brushing times, and toothpaste type, including whether toothpaste included antibiotics like triclosan, were not fully captured in the administered survey. These factors may have confounded our ability to appropriately identify an association between teeth brushing and the microbiome. In a similar vein, we are also missing insight into oral health, such as disease status or symptoms. Gathering this type of information can only be effectively executed by a dentist, which entails identifying a dental professional willing to go into the field to do so. While challenging, additional data about oral hygiene and oral health would provide insight into how these factors shift with the microbiomes of traditional populations.

Finally, we examined the oral-gut microbiome axis across traditional lifestyles. The oral and gut niches are linked by a consistent one-way flow of the saliva, food, and microbes from the mouth through to the colon. Although each niche hosts a distinct microbial community of locally adapted strains [117], the microbes originating in the oral cavity can colonize distal sites in the gut and the two communities have been demonstrated to be predictive of one another [118–120]. An estimated one third of oral microbes are able to colonize the guts of healthy individuals [121], with increasing rates of colonization in individuals with diseases such as bowel cancer [122, 123], rheumatoid arthritis [124], and inflammatory bowel diseases [125]. Given the higher incidence of these diseases in industrialized countries [126–128], an open question remains whether rates of translocation along the oral-gut axis vary with lifestyle, potentially being a risk factor for disease. While our 16S rRNA sequence data lacks the resolution needed to detect translocation specifically, we evaluated whether intra-individual similarity between the oral and gut microbiomes increased along the lifestyle gradient from traditional to agrarian. As expected under this hypothesis, there is a decrease in intra-individual Bray–Curtis distances along the gradient of traditional to agrarian populations within Nepal, although not statistically significant. This finding is similar to results from Cameroonian populations, in which the similarity of the oral-gut axis increased across a gradient of rural to urban populations, albeit not significantly [25]. Power analysis shows that given the effect size estimates at our current sample size, we are quite underpowered to detect a significant relationship, which may explain why there is a visible trend but no statistically significant trend. It is worth noting that we did not include Nepali expats or American industrialists in this analysis as we did not have paired oral-gut samples for those participants. Other analyses have suggested that the lifestyle gradient is subtle, meaning that detecting the trend between similar lifestyle stages is more challenging. As a result, without the inclusion of the industrialized populations, our ability to observe a significant trend may be dampened. We also note that the sequencing approach used only provides resolution to the genus level, so we were not able to distinguish whether similarity is a result of the translocation of oral strains to the gut versus a homogenization towards similar taxonomic profiles between the niches. Regardless, these results point towards an intriguing hypothesis: oral-gut microbial translocation increases with industrialization, potentially being a risk factor for disease. Future work would entail both increasing sample sizes of individuals across lifestyles as well as using methodologies that reliably track strain sharing across the two body sites.

While we identify new insights into the role of lifestyle in shaping the oral microbiome, there are limitations in our investigation. First, our sample sizes may seem low. While our sample size is sufficient to capture broad compositional differences across subsistence strategy groupings and differential abundance of common taxa, we may not have the ability to detect significant differences in lowly abundant or rare taxa. In practice, however, achieving large sample sizes in studies of traditional human lifestyles can be challenging. For example, the total population size of the Raute is only 550 individuals. We limited our sampling to unrelated individuals, further limiting the sample population. Similarly, the Chepang and Raji reside in small, remote villages scattered throughout Himalaya. As each village only consists of a few hundred individuals, it is very difficult to both reach these villages and also identify unrelated individuals. The industrialist sample size is also low; however, we note that the transition from traditional to agricultural lifestyles was the main focus of this study and that including publicly available data has the potential to introduce geographical and technical confounders. Ultimately, the purpose of this investigation is to examine the transitions across lifestyles, and as it stands, our study has sufficient sample size to identify novel statistical trends and address key gaps within the field. Additional sampling would allow us to dive deeper into these trends and better understand the specific contributors to this relationship.

Second, our investigation utilizes 16S rRNA sequencing, which only identifies taxa at genus-level resolution. As a result, we are unable to compare species or strain level differences in the microbiome across groups [129]. In addition, we currently can only indirectly infer the functional capabilities of specific microbes. While PICRUSt2 has been demonstrated to be more effective than other predictive functional potential methods, these tools are bound by their respective reference databases and have been demonstrated to miss microbial functional genes, highlighting the importance of validating such findings with metagenomic data [130, 131]. The collection of metagenomic data would allow for a more granular investigation of the microbial strains and functions that differ across lifestyles.

Third, our selection process of the lifestyle factors included in the CCA model may be biased. Our primary considerations were minimizing the number of tests performed to preserve power and minimizing multicollinearity, which is the correlation between predictor variables. Keeping collinear predictors can result in difficulty assessing the relationship between the predictors and outcome and therefore inaccurate estimations of model coefficients. Our method of predictor removal involved selecting variables based on contribution to lifestyle as our goal was to identify which lifestyle factors were associated with the microbiome; however, in doing so, we may have inadvertently removed any factors that are associated with the microbiome by means other than lifestyle. As a result, our CCA model may be inherently biased towards factors that follow the lifestyle trend and not fully capture all possible factors that influence the microbiome. It is for this reason that we also investigated the relationships of all lifestyle factors with the nine differentially abundant taxa to confirm our CCA findings. Future work should entail a more targeted investigation of the lifestyle factors with larger sample sizes to mitigate the concerns of loss of power due to multiple testing and multicollinearity.

## Conclusions

Our investigation of Nepali populations across a variety of human lifestyles expands our understanding of the oral microbiome at a global scale. In conjunction with gut microbiomes collected from the same individuals [15], we find that lifestyle is associated with the composition of both the gut and oral microbiomes, albeit to differing degrees. Metagenomic sequencing would provide finer-scale microbiome data that would allow us to more effectively identify the taxa and functional potential associated with lifestyle. In addition, studies in which industrialization is decoupled from background genetics, geography, and latitude will be essential for identifying the specific factors that result in microbiome differences across populations. Future work will reveal the extent to which oral microbiomes vary around the globe and refine our understanding of the environmental factors involved.

## Methods

### Ethics approval

This work was approved by the Ethical Review Board of the Nepal Health Research Council (NHRC) as well as by the Stanford University Institutional Review Board. Samples were collected between March and April 2016 with informed consent from all participants.

### Sample collection

Saliva samples were collected under informed consent using the DNA Genotek Oral Saliva kit (DNA Genotek, Stittsville, CA, USA) from five populations in Nepal: the Chepang (foragers, *n* = 19), Raji (recently settled/transitioned to agriculture, *n* = 11), Raute (recently settled/transitioned to agriculture, *n* = 14), Tharu (established agriculturalist, *n* = 21), and Newar ethnic groups (established agriculturalist, *n* = 8). Samples were collected in the winter of 2016 in March and April. Approximately 1 mL of unstimulated saliva samples were stored in the stabilizing buffer provided in the DNA Genotek kit and transported over 7–10 days at room temperature, after which they were stored at − 20 ºC for 2–3 months at the Institute of Medicine in Tribhuvan University, Kathmandu, Nepal.

At the time of saliva sample collection, detailed metadata was also collected from all participants. These data include demographic, anthropometric measurements, environmental, and dietary data using a survey questionnaire specifically designed for this study (Table S1). Participants’ responses to the survey data questionnaires were cleaned and standardized prior to analysis (Table S1).

Following the field work in Nepal, saliva samples were also collected from two populations in the USA with the same kits: the Newar population (expats emigrated from Nepal to the USA, *n* = 12) and European Americans (industrialist, *n* = 6) (Stanford IRB: 35,580). USA-based samples were collected in the winter of 2016 (November and December). All sampled individuals were unrelated and over 18 years old. Detailed survey data are available for the expat Newar population, but not the European American Industrialists (Table S1). Overall, our cohort included foragers (Chepang, *n* = 19), recently settled individuals (total, *n* = 25; Raji, *n* = 11; Raute, *n* = 14), established agriculturalists (total, *n* = 29; Tharu, *n* = 21; Newar, *n* = 8), expats (Newar, *n* = 12), and industrialists (European-Americans, *n* = 6).

### DNA extraction and 16S rRNA Amplicon sequencing

For samples collected in Nepal, total DNA was extracted at the Institute of Medicine (IOM) in Kathmandu using the Qiagen QIAamp MinElute Virus Spin kit (Qiagen, Germantown, MD, USA) according to the manufacturer’s protocol. Both the remaining original saliva samples and the Qiagen kit-extracted DNA were shipped to Stanford University on dry ice and then stored at either − 20 ºC until sequencing (extracted DNA) or at − 80 ℃ (remaining saliva sample). Total DNA was again extracted from saliva samples using the MO BIO PowerSoil DNA Isolation kit (MO BIO, Carlsbad, CA, USA) following the manufacturer’s recommended protocol. For samples collected in the USA, they were extracted with both the Qiagen QIAamp MinElute Virus Spin kit and MO BIO PowerSoil DNA Isolation kit in the US. Extraction-negative controls were included in all extractions to evaluate contamination during analysis.

The V4 hypervariable region of the 16S rRNA gene was amplified for all DNA extracts and PCR-negative controls using established primers and protocols [132]. The sample and negative control libraries were multiplexed and sequenced 250 bp single-end on the Illumina MiSeq platform at Stanford University, targeting a minimum of 25,000 reads per sample for accurate relative abundance quantification (Table S1).

### Sequencing data quality control and cleaning

All bioinformatic analyses were conducted in R version 4.1.2, unless otherwise stated. Single-end sequences were cleaned and processed using DADA2 v.1.22.0 [133]. First, reads were trimmed at 150 bp to remove low-quality bases, and then filtered to remove any reads with *N* nucleotides or more than two expected errors (max*N* = 0, maxEE = 2, truncQ = 2, Table S9). Next, sequence variants were inferred by pooling reads across samples (pool = TRUE). More specifically, 173 samples were pooled using 8,435,257 reads across 674,656 unique sequences. A sequence table was generated, consisting of 173 samples and 5068 amplicon sequence variants (ASV). Eleven percent of the reads were removed as chimeric, resulting in 7,490,294 reads and 1424 ASVs remaining (Table S9). ASVs were classified using the RDP v14 training set [134]. Finally, a phylogenetic tree was generated by performing multiple sequence alignment using DECIPHER v.2.22.0 [135] and then constructing the tree by using a neighbor-joining tree as a starting point via the package phangorn v.2.11.1 [136]. ASVs were then handed off to phyloseq v.1.38.0 for additional cleaning and downstream analysis [137].

Next, predicted contaminants were identified and removed via decontam v.1.14.0 using both the frequency and prevalence methods, thereby removing 19 ASVs [138]. Singletons and any taxa that do not appear at least five times across at least two samples were removed to account for spurious taxa stemming from sequencing errors (Table S9). For alpha diversity analyses, samples were rarefied using the rarefy_even_depth function from the phyloseq package by subsampling to 26,923 sequences (the minimum number of sequences across non-control samples) and then calculating alpha diversity from the rarefied samples (Fig. S19–S20). This was repeated 1000 times to account for randomness in rarefaction, and the mean alpha diversity value was calculated. Non-rarefied counts were used for tools with in-built compositionally aware transformation methods (i.e., centered log-ratio transformation). For all other analyses, counts were transformed to relative abundances using total-sum scaling. Log transformations were conducted with log10 and a pseudocount of 0.0001. Finally, individuals currently taking antibiotics were removed (*n* = 2) (Fig. S21), because antibiotics have previously been associated with changes in the oral microbiome [139]. Furthermore, current antibiotic use was found to be marginally significant when compared across Shannon alpha diversity for samples extracted with the Qiagen kit (*p* = 0.048, Kruskal–Wallis). The post-QC result sample sizes are 60 samples extracted by the PowerSoil kit and 69 samples extracted by the Qiagen kit (Fig. S22). The total number of ASVs across all samples extracted via the Qiagen and PowerSoil kits is 1000 ASVs.

### Random Forests classifier

Random Forests was first conducted by building 500 trees with 63 samples and 37 categorical variables from the survey data using the R package randomForest v.4.7–1.1 [140]. Random Forests models were subsequently conducted with 500 trees using microbiome data agglomerated to the genus level and transformed to relative abundances. Random Forests models were evaluated via out-of-bag error estimates, as well as assessing confusion matrices. Improvement beyond random chance was assessed using the R package verification v.1.42 [141].

### Diversity analyses

Five metrics were used to assess alpha diversity—Faith’s phylogenetic distance, Fisher’s alpha, Shannon alpha, Simpson alpha, and species richness [142–145]. Alpha diversity was calculated after rarefying sample counts to 26,923 counts 1000 times and taking the mean value. Kruskal–Wallis tests were performed to assess significant differences in alpha diversity between the ethnic groups. Dunn’s post hoc test was used to identify the group that was driving the differences.

Beta diversity was calculated from relative abundance counts using Bray–Curtis distance, unweighted Unifrac, and weighted Unifrac [40, 146]. The resulting distances were ordinated using PCoA as implemented in phyloseq. PERMANOVA was performed to assess dissimilarity between ethnic groups using vegan v.2.6–4, permuted 99,999 times [147, 148]. Pairwise PERMANOVA comparisons were conducted using pairwiseAdonis v.0.4.1 [149]. Jonckheere-Terpstra tests were used to assess whether the individual PCoA axes followed the lifestyle trend [150].

### Extraction kit comparison

The Qiagen QIAamp MinElute Virus Spin and MO BIO PowerSoil DNA isolation extraction kits were compared based on overall microbiome beta diversity. Beta diversity was calculated, and PERMANOVA was performed as described above. Comparisons suggest that while there is some qualitative difference between the two kits, there is no statistically significant difference (PERMANOVA, *p* > 0.05) (Fig. S3A), and PCoA axes 1 and 2 are highly correlated across kit (PCoA1 rho = 0.96, *p* < 2.2*10^−16^; PCoA2 rho = 0.89, *p* < 2.2*10^−16^) (Fig. S3B). For fidelity, diversity analyses were conducted using both extraction kits, but other analyses were conducted using only the dataset extracted via the Qiagen kit, selected for its larger sample size (Fig. S3).

### Differential abundance analysis

Differential abundance analysis was conducted with microbiome count data agglomerated to the genus level using the ALDEx2 v.1.29.2.1. The Kruskal–Wallis module was used to identify microbes differentially abundant across all lifestyles, whereas the standard *t* test module was used for assessing microbes differentially abundant across two conditions. To account for multiple tests, the Benjamini–Hochberg method for *p*-value correction was applied [151]. Effect sizes > 1 and adjusted *p*-values < 0.05 were considered significant.

To validate that our findings are robust to the inclusion of sex and age as covariates, the GLM module was used. Because the outputs for the two modules differ, the data was reanalyzed with and without covariates for comparison purposes. Furthermore, because we are missing age data for the American industrialists, this comparison was conducted twice: once with all individuals and sex as the only covariate, and once with the Nepali individuals and both sex and age as the covariates. To account for multiple tests, the Benjamini–Hochberg method for *p*-value correction was applied [151]. Adjusted *p*-values < 0.05 were considered significant.

To identify which microbes followed the lifestyle trend, Jonckheere-Terpstra tests were performed for each genus using microbiome data agglomerated to the genus level and transformed to relative abundances [150]. All *p*-values stemming from the Jonckheere-Terpstra tests were corrected for multiple tests using the Benjamini–Hochberg method [151], and adjusted *p*-values < 0.05 were considered significant.

To validate that the trend observed is not an artifact of sequencing depth, read depth was examined across lifestyles. Sequencing depth was not found to be associated with lifestyle (*p* > 0.05, Kruskal-Wallis; Fig. S23 A). For additional confirmation, *Brachymonas* relative abundance was assessed against read depth, and no correlation was found (*p* > 0.05, rho =  − 0.11, Spearman correlation; Fig. S23 B).

### Associations between lifestyle factors, population, and the microbiome

Correspondence analysis (CA) was performed on the survey metadata using FactoMineR v.2.9 [152]. To determine whether the samples (rows) and lifestyle factors (columns) were significantly associated, a chi-square test of independence was performed. The top 10 most contributing factors for CA1 and CA2 were identified. Alpha and beta diversity values were calculated as described above, and correlations to the CA axes were calculated using Spearman correlation. Canonical correspondence analysis (CCA) was performed with microbiome data agglomerated to the genus level, transformed to relative abundances, and then log-transformed using vegan v.2.6–4 [148]. As there was multicollinearity between the 37 metadata variables (as established by variance inflation factor analysis using the function vif.cca, VIF > 10), we considered the top 10 categorical lifestyle factors contributing to CA1 or CA2 only to reduce this burden. As some factors were top contributors to both CA1 and CA2, a total of 15 lifestyle factors were used for the CCA model. These 15 factors did not demonstrate collinearity (VIF < 10). Model significance and significant lifestyle factors were identified and assessed using the function anova.cca from the vegan package, which performs an ANOVA-like permutation test, permuted 999 times.

To identify which lifestyle factors were specifically associated with a particular taxon, a linear model was generated for the relationship between lifestyle variables and a selected taxon and then tested for significance. Taxa were agglomerated to the genus level, transformed to relative abundance, and then log-transformed for visualization. All *p*-values stemming from linear models were corrected for multiple tests using the Benjamini–Hochberg method [151], and adjusted *p*-values < 0.05 were considered significant. To test whether alcohol use and smoking follow the lifestyle gradient from traditional to expatriate, we performed the Cochran-Armitage test using the function prop_trend_test from the package rstatix 0.7.2 [153].

### PICRUSt2 analysis

PICRUSt2 v2.5.2 was conducted using the “–per_sequence_contrib” option to predict pathway abundances for each ASV [42]. Pathway abundances were predicted using the KEGG database FTP release 2022–11-07 [154]. Differential abundance analysis and examination of functions following the lifestyle trend were conducted as previously described using the pathway abundance output. To investigate microbiome functional enrichment, differential abundance analysis was conducted with the KEGG Orthologs (KO) and any significant K genes (prior to multiple test correction) were inputted into MicrobiomeProfiler v1.0.0 [155].

### Network analysis

Network analysis was conducted using the SparCC module in SpiecEasi v.1.1.2 [44, 45]. Microbiome count data agglomerated to the genus level was inputted to generate correlations between taxa. Networks were analyzed for centrality, modularity, and degree distribution using igraph v.1.6.0 [156]. Co-abundance groups (CAGs) were generated using the cluster_fast_greedy function in the igraph package [157]. Networks were plotted in Cytoscape v.3.8.2 [156, 158]. Nodes with 0 edges were removed for visualization and generating CAGs.

### Comparison of the gut and oral microbiomes

The gut and oral microbiomes were only compared between the same individuals overlapping across both studies (Table S7). PCoA axes were generated for the microbiome datasets for each location as previously described and Spearman correlations were calculated between axes. To compare intra-individual oral-gut microbiome dissimilarity across lifestyles, Bray–Curtis distance was calculated between the gut and oral microbiomes within each individual and then compared across all individuals for differences across lifestyle, with significance determined by a Jonckheere-Terpstra test. To conduct the power analysis, Cohen’s D was calculated between each group and used as effect sizes. Power was subsequently calculated using the package clinfun v.1.1.5 [159].

## Supplementary Information

Additional file 1: S1 Figure: Confusion matrix of random forest classification based on survey data. S2 Figure: Correspondence analysis based on survey data. S3 Figure: Microbiome composition within sample is similar across the two extraction kits tested. S4 Figure: All alpha diversity metrics across lifestyle groups with DNA extracted using the Qiagen kit. S5 Figure: Alpha diversity metrics with DNA extracted using the PowerSoil kit. S6 Figure: Oral microbiome composition extended figures—Axis 2 from Qiagen kit extraction, and ordinations on samples extracted using the PowerSoil kit. S7 Figure: Oral microbiome composition calculated with unweighted Unifrac distance. S8 Figure: Oral microbiome composition calculated with weighted Unifrac distance. S9 Figure: Confusion matrix of Random Forest classification based on microbiome data. S10 Figure: Correlations between diversity metrics and CA axes. S11 Figure: Smoking and *sisnu* are associated with several differentially abundant taxa. S12 Figure: Alcohol and Smoking by lifestyle. S13 Figure: Predicted functional abundance significantly differs by lifestyle. S14 Figure: Metabolism pathways form the majority of the significantly differentially abundant predicted functions from PICRUSt2. S15 Figure: Microbiome functional enrichment analysis. S16 Figure: *Fusobacterium* contributes to predicted platinum resistance, which varies by lifestyle. S17 Figure: Correlations between the top three oral and gut microbiome PCoA axes. S18 Figure: Power analysis for the Jonckheere-Terpstra test used in the oral-gut dissimilarity analysis. S19 Figure: Rarefaction curves. S20 Figure: Read depth across samples after read QC. S21 Figure: Comparison of antibiotic use on oral microbiome diversity. S22 Figure: Final sample size per lifestyle per extraction kit. S23 Figure: Read depth against lifestyle and *Brachymonas* relative abundance.

Additional file 2: S1 Table—Sequence, survey, population, and questionnaire info of the sampled individuals. Table S1 describes survey and sequence metadata data collected. Column abbreviations and responses are explained in greater detail in Tab 3. Column names that end with “2” contain responses that were categorized and transformed to a scale of 0-3, in which possible values for binary variables are 0 or 3 (ie. sex) and possible values for continuous variables are 0, 1, 2, or 3 (ie. fuel source). No survey data was collected for the American Industrialists. Tab 2 describes the lifestyle pertaining to each population and their sample sizes. Tab 3 contains the survey questionnaire, including the codes pertaining to each question asked and list of possible responses.

Additional 3: S2 Table—Oral microbiome differential abundance results from ALDEx2. Oral microbiome genera tested for differential abundance across lifestyle. Overall, 2/111 genera were identified as significantly differentially abundant. Kruskal-Wallis module was utilized and *p*-value correction was applied using the Benjamini-Hochberg method. Both unadjusted (kw.ep) and adjusted *p*-values (kw.eBH) are shown, and adjusted *p*-value < 0.05 is the threshold for significance.

Additional file 4: S3 Table—Oral microbiome differential abundance results from ALDEx2 with covariates age and sex. Oral microbiome genera tested for differential abundance across lifestyle with age and sex as covariates. Due to the lack of age data for the American Industrialists, only sex was included in the model tested with all individuals, whereas both sex and age were included in the model tested across the Nepali individuals. The glm module was utilized and *p*-value correction was applied using the Benjamini-Hochberg method. Both unadjusted (kw.ep) and adjusted *p*-values (kw.eBH) are shown, and adjusted *p*-value < 0.05 is the threshold for significance. Tab 1 shows the output for the glm model with no covariates and all individuals. Tab 2 shows the output for the glm model with sex as a covariate and all individuals. Tab 3 shows the output for the glm model with no covariates and only Nepali individuals. Tab 4 shows the output for the glm model with both sex and age as covariates and only Nepali individuals.

Additional file 5: S4 Table—Results of genera tested for following the lifestyle gradient. All genera tested for following the lifestyle gradient using the Jonckheere-Terpstra test followed by the Benjamini-Hochberg method to correct for multiple tests (BHadj_*p*_value). Adjusted *p*-value < 0.05 is the threshold for significance. Nine genera significantly follow the lifestyle gradient. 

Additional file 6: S5 Table—Associations between differentially abundant microbes and lifestyle factors. Associations between differentially abundant microbes from the Jonckheere-Terpstra test and lifestyle factors were tested via linear models. Linear models were generated between each microbe and each lifestyle factor and then tested for significance, for a total of 333 tested associations. P-value correction was applied using the Benjamini-Hochberg method. Both unadjusted and adjusted *p*-values are shown. Adjusted *p*-value < 0.05 is the threshold for significance.

Additional file 7: S6 Table—Predicted functional potential differential abundance results. PICRUSt2 predicted functions were analyzed for differential abundance based on lifestyle. None of the 107 tested functions were found to be significant after multiple test correction, but 21/107 pathways were significant prior to correction. Kruskal-Wallis module in ALDEx2 was utilized and p-value correction was applied using the Benjamini-Hochberg method. Both unadjusted (kw.ep) and adjusted *p*-values (kw.eBH) are shown. Adjusted *p*-value < 0.05 is the threshold for significance.

Additional file 8: S7 Table—Samples overlapping between the gut and oral microbiome studies. List of samples overlapping between the gut and oral microbiome studies, along with the samples unique to each microbiome study. The first column “both” lists sample IDs that are associated with both gut and oral samples. The second column “gut_only” lists sample IDs that are associated with only gut samples. The third column “oral_only” lists sample IDs that are associated with only oral samples.

Additional file 9: S8 Table—Gut microbiome differential abundance results from ALDEx2.  Gut microbiome genera analyzed for differential abundance based on lifestyle via ALDEx2. Overall, 37/136 genera were identified as significantly differentially abundant. Kruskal-Wallis module was utilized and *p*-value correction was applied using the Benjamini-Hochberg method. Both unadjusted (kw.ep) and adjusted *p*-values (kw.eBH) are shown. Adjusted *p*-value < 0.05 is the threshold for significance.

Additional file 10: S9 Table—Read counts through each sequence processing step. Read counts at each step of sequence processing, starting with raw demultiplexed reads and all the way through DADA2, merging, and chimera removal. “Input” column refers to the number of raw reads obtained per sample after sequencing, “filtered” refers to the number of reads remaining after initial read QC, “denoised” refers to the number of reads remaining after denoising in DADA2, “nochim” refers to the number of reads remaining after chimeric sequences were removed, and “retained_overall” is the total proportion of reads retained following all QC steps from the input amount. Table does not include the samples that failed to pass initial read QC.

## Data Availability

Sequence data can be retrieved from the Sequence Read Archive (SRA) under BioProject number PRJNA1098228. All scripts used in this analysis are available at http://github.com/davenport-lab/Nepali_oral_microbiomes.

## Abbreviations

CA: Correspondence analysis
PERMANOVA: Permutational multivariate analysis of variance using distance matrices
PCoA: Principal coordinate analysis
CCA: Canonical correspondence analysis
ANOVA: Analysis of variance
padj: *p*-Values adjusted for multiple test correction
CAG: Co-abundance group
USA: United States of America
IRB: Institutional Review Board
bp: Base pair
ASV: Amplicon sequence variants
KO: KEGG Orthologs
