# Correction: Nepali oral microbiomes reflect a gradient of lifestyles from traditional to industrialized

**DOI:** 10.1186/s40168-024-01988-6

**Published:** 2024-12-03

**Authors:** Erica P. Ryu, Yoshina Gautam, Diana M. Proctor, Dinesh Bhandari, Sarmila Tandukar, Meera Gupta, Guru Prasad Gautam, David A. Relman, Ahmed A. Shibl, Jeevan Bahadur Sherchand, Aashish R. Jha, Emily R. Davenport

**Affiliations:** 1 Department of Biology, Pennsylvania State University, University Park, PA USA; 2 Genetic Heritage Group, Program in Biology, New York University Abu Dhabi, Abu Dhabi, UAE; 3 Department of Microbiology and Molecular Genetics, McGovern Medical School, The University of Texas Health Science Center at Houston, Houston, TX USA; 4 Public Health Research Laboratory, Institute of Medicine, Maharajgunj, Kathmandu, Nepal; 5 School of Public Health, University of Adelaide, Adelaide, SA Australia; 6 Organization for Public Health and Environment Management, Lalitpur, Bagmati Nepal; 7 Sidney Kimmel Medical College, Philadelphia, PA USA; 8 Department of Geography, Tribhuvan University, Nepalgunj, Nepal; 9 Department of Medicine, Stanford University, Stanford, CA USA; 10 Department of Microbiology and Immunology, Stanford University, Stanford, CA USA; 11 Section of Infectious Diseases, Veterans Affairs Palo Alto Health Care System, Palo Alto, CA USA; 12 Center for Genomics and Systems Biology, and Public Health Research Center, New York University Abu Dhabi, Abu Dhabi, UAE; 13 Huck Institutes of the Life Sciences, Pennsylvania State University, University Park, PA USA

**Correction** **:** **Microbiome 12, 228 (2024)**

**https://doi.org/10.1186/s40168-024-01941-7**

Following publication of the original article [1], the author reported that the country for Afilliation 7 should be USA and not UAE.

7 Sidney Kimmel Medical College, Philadelphia, PA, UAE.

Should be:

7 Sidney Kimmel Medical College, Philadelphia, PA, USA.

The original article has been updated.
